# Development and preliminary validation of the Arabic Quality of Life During War Scale (AQOL-War)

**DOI:** 10.1371/journal.pone.0358784

**Published:** 2026-10-29

**Authors:** Mohamed Fakhry Hussein, Yasmine Yousry Mohamed, Basma Tolba, Suzan Abdel-Rahman, Mahmoud A. Abdel-Fattah, Nesma Gamal Elsheikh, Nehal Elkholy, Assem Gebreal, Ayoub Ali Alshaikh, Alaa Yahia Fadle Modawy, Omelhassan Abdelgader Alawad, Randa Bushra Ibrahim Elbushra, Ramy Mohamed Ghazy

**Affiliations:** 1 Department of Occupational Health and Industrial Medicine, High Institute of Public Health, Alexandria University, Alexandria, Egypt; 2 Department of Family Health (Maternal and Child Health), High Institute of Public Health, Alexandria University, Alexandria, Egypt; 3 Department of Neuropsychiatry, Faculty of Medicine, Ain Shams University, Cairo, Egypt; 4 Department of Biostatistics and Demography, Faculty of Graduate Studies for Statistical Research, Cairo University, Cairo, Egypt; 5 Department of Applied Statistics and Econometrics, Faculty of Graduate Studies for Statistical Research, Cairo University, Giza, Egypt; 6 Department of Geriatric Medicine, Faculty of Medicine, Ain Shams University, Cairo, Egypt; 7 Department of Geriatric Medicine, Electricity Hospital, Cairo, Egypt; 8 Faculty of Medicine, Alexandria University, Alexandria, Egypt; 9 Department of Family and Community Medicine, College of Medicine, King Khalid University, Abha, Saudi Arabia; 10 Department of Pharmaceutical Biotechnology, Al Mughtaribeen University, Sudan International University, Khartoum, Sudan; 11 Faculty of Medicine and Health Sciences, Omdurman Islamic University, Khartoum, Sudan; 12 Ibrahim Malik Hospital, Port Sudan, Sudan; 13 Department of Tropical Health, High Institute of Public Health, Alexandria University, Alexandria, Egypt; Universiti Sains Malaysia - Kampus Kesihatan, MALAYSIA

## Abstract

**Background:**

War profoundly affects quality of life (QOL) by disrupting daily routines, damaging infrastructure, and creating fear and uncertainty**.** This study aimed to develop and preliminarily validate the Arabic Quality of Life During War Scale **(**AQOL-War).

**Methods:**

This cross-sectional study was conducted in Sudan during wartime from June 11 to September 10, 2024. Data were collected through an anonymous online survey and face-to-face interviews. Cronbach’s alpha was used to measure the tool’s internal consistency. We assessed face, content, concurrent, and structural validity, along with measurement invariance.

**Results:**

Among 635 participants, the mean age was 27.9 ± 9.1 years, 72.1% were females, 65.4% lived in urban areas, 67.1% lived in their own house, 71.5% were single, 70.1% had a university education, 71.7% were not currently employed, 19.2% were from Khartoum State, and 21.3% reported losing a family member during the recent war. Exploratory factor analysis yielded a Kaiser-Meyer-Olkin (KMO) of 0.889, and Bartlett’s test was significant (χ² = 4965.76, p < 0.001). Three factors (environment and security, psychological health, and physical health) with eigenvalues > 1 were extracted with a cumulative variance explained of 41.8%. Confirmatory factor analysis showed satisfactory model fit (χ2 = 831.36, p < 0.001) with good fit indices [root mean square error of approximation (RMSEA) = 0.06, root mean square residual (RMR) = 0.04, comparative fit index (CFI) = 0.916, goodness of fit index (GFI) = 0.936, Tucker-Lewis Index (TLI) = 0.905, and non-normed fit index (NNFI) = 0.905]. The overall Cronbach’s alpha for the quality-of-life tool was 0.93; values for “environment and security”, “psychological health”, and “physical health” were 0.94, 0.84, and 0.83, respectively.

**Conclusions:**

The AQOL-War demonstrated satisfactory preliminary validity, reliability, and configural and metric invariance. It may provide a contextually relevant measure for researchers and clinicians working with Arabic-speaking populations affected by war. Further cross-context replication and longitudinal evaluation are needed.

## Introduction

Quality of life (QOL) is a multidimensional concept that encompasses overall well-being and negative aspects of life. It reflects how individuals perceive their position in the context of their culture, goals, expectations, and standards. This subjective experience is shaped by physical and mental health, social relationships, environmental conditions, economic circumstances, and exposure to armed conflicts and wars [1–3].

War is a pervasive feature of human history: currently, over 110 armed conflicts are active worldwide [4]. War exerts a deep and destructive influence on QOL by upending everyday life, damaging essential infrastructure, and generating fear and unpredictability [5]. Physical and psychological trauma, economic hardship, displacement, and bereavement collectively produce a significant decline in well-being [6,7]. The devastating effects of war extend far beyond combat-related fatalities, impacting economies, health, and QOL among populations living in war zones, as well as displaced persons and refugees [8,9].

The war between the Sudanese Armed Forces and the Rapid Support Forces, which erupted in April 2023, illustrates this burden acutely. Sudan now faces one of the world’s fastest-evolving humanitarian disasters: approximately 25 million people, including over 14 million children, require urgent assistance; famine threatens more than one-third of the population, and over 8.6 million people have been internally or externally displaced, constituting the world’s largest displacement crisis [6,7].

To refine the theoretical framework of this study, it is essential to distinguish between general well-being, which often assumes a baseline of environmental stability, and “survival-based” QOL, which emerges under the chronic stress of war. While traditional models often view QOL as a relatively static measure of satisfaction, the transactional model proposes that QOL is a dynamic process shaped by the interaction between individual attributes and environmental opportunities [10]. In combat settings, this “transaction” is severely restricted; the environment no longer facilitates growth but instead demands a focus on fundamental physical and psychological resilience. This shift necessitates a more holistic understanding of trauma, as evidenced by Chaudhari et al. [11], who demonstrate that in forcibly displaced populations, effective QOL recovery requires integrated interventions that simultaneously address physical somatic symptoms and psychological distress. By recognizing QOL in these contexts as a struggle for functional agency rather than a pursuit of life satisfaction, researchers can better capture lived experiences in the Arab region and other understudied war-affected settings.

In a stable society, QOL domains such as “environment” and “security” often exist as distinct constructs, one referring to physical surroundings and the other to personal safety [12]. However, under the conditions of full-scale armed war, these domains undergo a conceptual convergence. The environment ceases to be a background setting and becomes the primary source of threat; thus, “living environment” and “security” merge into a single, cohesive **“**Ecological safety**”** factor [13]. Our operational model proposes that war-affected QOL is a reflective construct manifested through three specific dimensions: physical health (survival and somatic resilience), psychological health (emotional stability and coping), and environment and security (the integration of physical safety and habitat stability). We justify this three-domain structure based on the World Health Organization Quality of Life-BREF (WHOQOL-BREF) questionnaire and the hierarchy of needs in crisis: when the environment is weaponized, the individual’s QOL is no longer a measure of elective satisfaction, but a reflective index of their ability to maintain physical and mental integrity while navigating a high-risk landscape [14].

The development of a new instrument is necessitated by a measurement gap in established, peacetime QOL scales. While instruments like the WHOQOL-BREF [15], the Arabic version of the 12-item Short-Form (SF-12) health survey questionnaire [16], and the Manchester Short Assessment of Quality of Life (MANSA) [17] are highly effective in stable or clinical settings, they fail to achieve ecological validity in active combat zones due to construct underrepresentation. For example, WHOQOL-BREF’s environmental domain focuses on aesthetics, physical safety in a civil sense, and transportation, factors that are fundamentally redefined when the environment becomes weaponized. In a war context, “satisfaction with transport” is superseded by “freedom of movement” and “evacuation feasibility” [15]. Similarly, the MANSA focuses on satisfaction with social life and employment, which may be entirely disrupted by mass displacement and economic collapse [17]. Finally, the SF-12 is too short and cannot capture war-specific situations [16].

The absence of a validated Arabic questionnaire for QOL for combat settings is a significant problem, considering the substantial Arab population of approximately 492 million across 22 countries and the region’s frequent wars [18]. This lack of a reliable tool hinders the ability to accurately measure and understand the profound impact of war on the well-being of Arab communities. Guided by the need for a theoretically grounded assessment, this study is the first to examine the underlying factor structure of a newly developed scale to determine its alignment with the proposed three-domain model. Furthermore, it evaluated the scale’s psychometric stability, specifically its internal consistency, construct validity, and measurement invariance across diverse populations within the context of war. Consequently, this study aimed to develop and validate the Arabic Quality of Life During War Scale (AQOL-War) as a foundational instrument for researchers and healthcare professionals working with populations affected by war.

## Methodology

### Study design and setting

An anonymous cross-sectional study was conducted to assess QOL during wartime from June 11 to September 10, 2024. The questionnaire was developed and administered electronically using Google Forms and distributed throughout Sudan via widely used social media platforms, including Facebook, WhatsApp, and Twitter, as well as email, yielding 544 responses. In areas with limited or no internet access, data were collected through face-to-face interviews using the same questionnaire and later entered into the electronic database by the researcher when an internet connection became available (91 responses).

To ensure the safety of both participants and researchers in high-risk zones during face-to-face interviews, data collection was conducted by trained local personnel who maintained ongoing communication with designated security coordinators to monitor changes in the security situation and the risk of war escalation. Interviews were held only in secure settings such as designated community hubs or facilities operated by humanitarian organizations. Consistent with ‘do no harm’ ethical guidelines, data collectors were instructed to immediately suspend or postpone an interview if security conditions deteriorated or if their presence was deemed to increase the risk to the participant or research team. Furthermore, to minimize participants’ exposure to potentially hazardous conditions, the questionnaire was specifically kept concise and interviews were conducted as efficiently as possible, limiting the time participants needed to remain in potentially vulnerable settings.

### Study participants

Participants were eligible if they were adult individuals aged 18 years or older, held Sudanese nationality, were residing inside Sudan during the ongoing war, and were able to understand and respond to the Arabic-language questionnaire. Moreover, for online participation, participants required access to the internet and a smartphone or computer. Participants recruited through face-to-face interviews were not required to have internet access. Individuals were excluded if they were unable to provide informed consent due to severe cognitive impairment, acute psychosis, or other conditions impairing decision-making capacity. Individuals who were severely ill or injured were also excluded when participation was considered likely to pose a safety risk or impose an undue physical or psychological burden.

### Sample size and sampling method

For exploratory factor analysis (EFA), a total of 350 participants were recruited, based on the recommended ratio of 10 participants per item, given the questionnaire consists of 35 items [19]. For subsequent confirmatory factor analysis (CFA) using structural equation modeling (SEM), an additional independent sample of 285 participants was required based on predetermined parameters of significance level (α) = 0.05, power = 0.95, effect size = 0.3, three latent variables, and 35 observed variables [20]. The total sample of 635 participants was randomly divided into an EFA sample (n = 350) and an independent CFA sample (n = 285). Participants were recruited using convenience and snowball sampling methods.

### Questionnaire development and validation

#### Item generation and formatting.

To develop the questionnaire, the researchers followed a systematic, multi-stage procedure to ensure maximum content validity. Initially, an item pool was generated through a deductive review of existing QOL instruments and literature regarding the psychosocial impact of war in the Arab region [16,17,21–25]. Items were selected based on three strict criteria: (1) Contextual relevance, ensuring items captured war-specific stressors like displacement and ecological safety; (2) Linguistic accessibility, ensuring the phrasing was appropriate and comprehensible for speakers of the Sudanese Arabic dialect; and (3) Non-redundancy, to minimize duplication and maintain a parsimonious scale.

To ensure comprehensive coverage of all aspects of the survival-based QOL construct, the initial pool was subjected to an inductive refinement phase via a panel of six experts in public health, environmental health, QOL, internal medicine, family medicine, and mental health. These experts reviewed the pool of questions against the proposed theoretical domains: environment and security, psychological health, and physical health, and assessed their relevance, clarity, and comprehensiveness to establish face and content validity (Fig 1). Existing questionnaires in the field were examined, and experts provided feedback on item wording and clarity.

**Fig 1:**
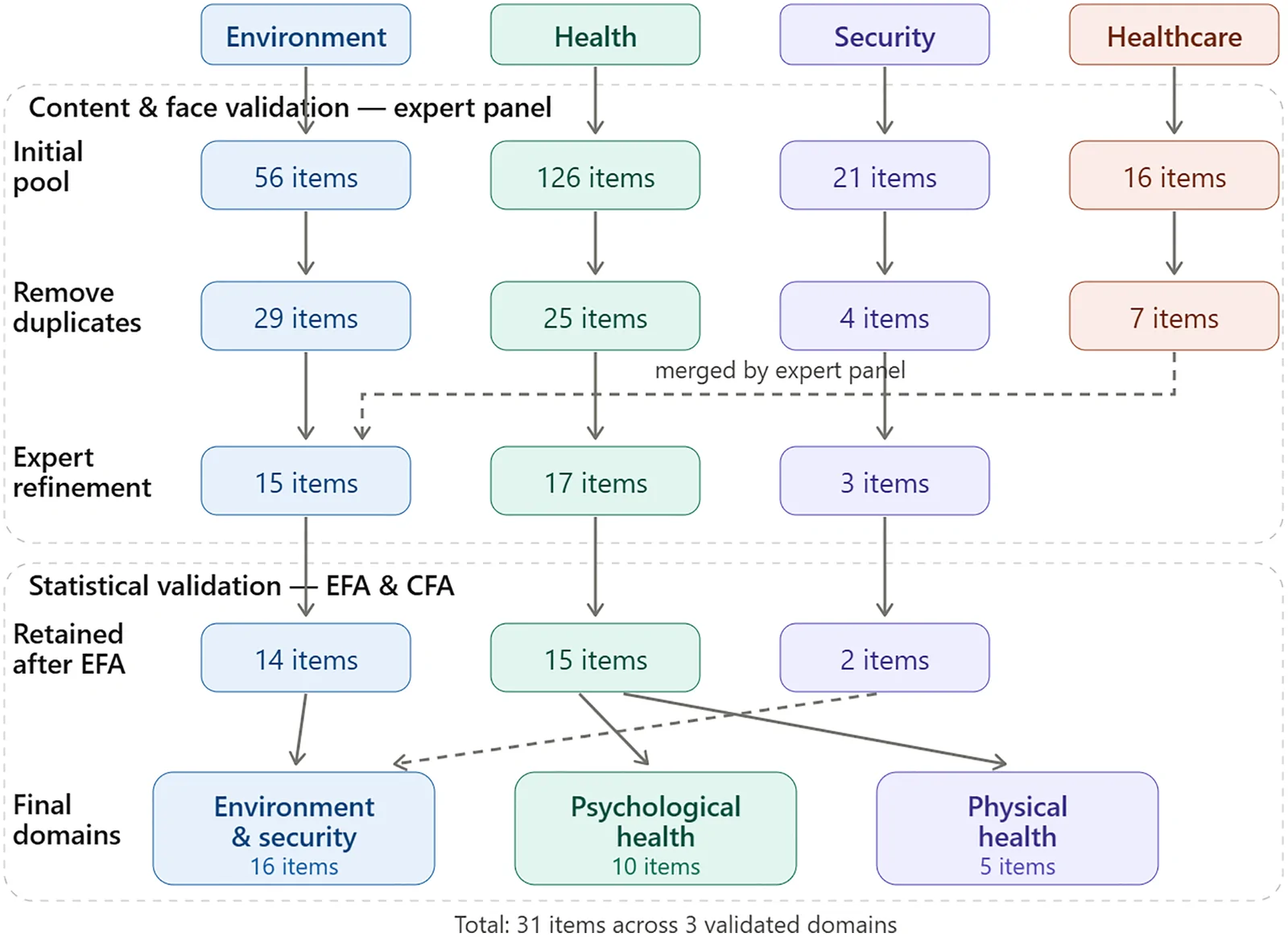
Item development and validation pathway for the AQOL-War. Items underwent content and face validation, including expert-panel review, duplicate removal, and refinement against the proposed domains, followed by statistical validation using exploratory and confirmatory factor analysis. The final instrument comprised three validated domains and 31 items.

Based on the experts’ feedback, items were revised to improve clarity and eliminate ambiguity, technical terminology, and unnecessary complexity. Particular attention was given to ensuring that items were clear, concise, unambiguous, and free from double-barreled questions and negatively worded constructs.

#### Content validity.

The content validity index (CVI), a metric used to assess the relevance and representativeness of items within a questionnaire, was evaluated by experts. Calculating the CVI involves two levels: the item-level CVI (I-CVI) and the scale-level CVI (S-CVI). The I-CVI was calculated as the proportion of experts who rated an item as either 3 (quite relevant) or 4 (highly relevant) on a four-point relevance scale, divided by the total number of experts (n = 6).

The S-CVI provides an overall index of content validity for the entire scale, capturing the relevance of all items. The S-CVI was calculated through S-CVI/Ave (average), which averages the I-CVI scores across all items. The S-CVI/Ave was 0.85 for the environment domain, 0.80 for the health domain, and 0.78 for the security domain, indicating satisfactory CVI. The overall S-CVI/Ave was 0.82, demonstrating satisfactory content validity. The domain-level findings indicated that most items were rated by the expert panel as relevant and appropriate for their respective constructs (Table A1 in S3 File).

#### Pilot test (cognitive interviews).

A pilot study was conducted among a convenience sample of 60 participants residing in war-affected regions of Sudan to assess the tool’s feasibility, comprehensibility, and acceptability. The pilot study also assessed the approximate time required to complete the questionnaire, which was approximately 15 minutes, and identified potential difficulties in understanding or interpreting individual items. Several items were modified to resolve semantic ambiguity and ensure that the questions addressed the specific conditions of the war in Sudan. For example, we clarified the definition of “basic needs” by explicitly listing (food/water/medicine/clothes) to minimize subjective interpretations and extended the context of transportation by adding “(even for emergencies)” to include routine mobility and critical evacuation needs. To ensure technical clarity, we added the term “(illumination)” to the item regarding neighborhood lights and broadened the terminology for medical access to include “(facilities/shelters),” acknowledging the informal and temporary nature of healthcare points in active combat zones. Finally, the phrasing was expanded to “aggressive/violent behavior” because pilot participants distinguished between normalized wartime aggression and overt violence, both of which significantly impact QOL.

### Data collection tool

The survey comprised four sections, beginning with a consent question to participate in the study (S1 File).

- The first section collected socio-demographic data such as age, gender, nationality, place of residence (rural or urban), state of residence, housing type, marital status, education level, current occupation (including pre-war occupation if unemployed), household size, family status (together or separated), and any family losses due to the war.
- The second section consisted of 16 questions assessing the environment and security during the war. The environment part (14 questions) included items assessing overall life satisfaction, satisfaction with the availability and accessibility of humanitarian aid, basic needs, healthcare services, water sources, nighttime lighting, transportation, work, education, energy, and communication services, besides the degree of environmental pollution. In addition, security concerns during the war (two questions) asked about the safety of the participants’ living area and whether there were places that women avoided.
- The third section assessed psychological health (10 questions), including questions about the impact of war on overall health, sleep patterns, psychological well-being, emotional states (sadness, fear, anger, and violence), coping abilities, and family and community relationships.
- The fourth section addressed the physical health items (five questions) which included questions about physical injuries or disabilities the participants or their family members were exposed to, and the exacerbation of pre-existing and chronic conditions.

The final version of the scale comprised 31 questions. A five-point Likert scale ranging from “extremely dissatisfied” to “extremely satisfied” was used for the environment section. Security concerns were assessed using a five-point Likert scale ranging from “extremely unsafe” to “extremely safe.” Psychological and physical health were evaluated using a five-point Likert scale ranging from “extremely affected by war” to “extremely not affected,” while frequency items ranged from “rarely” to “most of the time”. The total score ranged from 31 to 155, with higher scores indicating better QOL. To ensure careful reading and response variability, some questions were reverse scored (H10, H12, H13, H14, H16, and H17). These reverse-scored items were subsequently adjusted to maintain consistency with the overall positive scoring direction.

### Psychometric analysis

#### Reliability and item analysis.

Cronbach’s alpha was used to measure the tool’s internal consistency. An alpha coefficient of ≥0.70 was considered acceptable for research purposes, with higher values indicating greater internal consistency [26]. Split-half reliability was estimated using both the Güttman split-half coefficient and the Spearman–Brown prophecy formula. The AQOL-War was divided into two equivalent halves, and the correlation between the two half-scores was calculated. The Spearman–Brown formula was then applied to correct this correlation and estimate full-scale reliability. The Guttman coefficient provided an independent lower-bound reliability estimate that does not assume equal variance between halves [19,27]. Composite reliability (CR) was assessed to evaluate the internal consistency and reliability of the latent constructs identified in the questionnaire. CR values were calculated for the three latent variables based on the standardized factor loadings obtained from the CFA.

#### Validity of the questionnaire.

The validity of the questionnaire was assessed using multiple complementary approaches: convergent validity, discriminant validity, concurrent validity, structural validity through EFA and CFA, and known-groups validity.

**Convergent validity**: Convergent validity was assessed primarily by using the average variance extracted (AVE) and standardized factor loadings obtained from the CFA. An AVE ≥ 0.50 was considered indicative of adequate convergent validity, suggesting that the latent construct explained at least 50% of the variance in its observed indicators [27]. Standardized factor loadings were also analyzed to assess how well each individual item reflected its corresponding latent construct.

Inter-item and item-total correlations were additionally examined as indicators of item homogeneity and internal consistency within each domain. Pearson correlation coefficients were calculated to assess the relationships among items within the same domain and between individual items and their corresponding domain scores.

**Discriminant (Divergent) validity**: Discriminant validity was assessed by analyzing the correlation matrix of the three latent constructs of the AQOL-War framework. The correlations between constructs were evaluated to determine whether the domains represented conceptually distinct dimensions of QOL. In addition, the Fornell–Larcker criterion was applied to confirm discriminant validity. Under this criterion, the square root of the AVE for each construct must exceed its correlations with all other constructs. This demonstrates that each construct explains more variance in its own indicators than in the indicators of different constructs [27].

**Concurrent validity**: It was assessed by administering the Arabic version of the 12-Item Short Form Health Survey (SF-12) [28] alongside the newly developed AQOL-War. The AQOL-War comprised 35 items covering the domains identified during the scale-development process (before final validation): 15 items related to environment, 3 to security, 12 to psychological health, and 5 to physical health. The SF-12 was used as an established measure of health-related QOL to examine the extent to which AQOL-War scores were associated with a validated measure of general health-related QOL. The SF-12 consisted of 12 questions to assess eight health-related domains: physical activity, physical role functioning, pain, general health, vitality, social activity, mental health, and emotional role functioning. All items were closed-ended, providing multiple-choice options. The Arabic SF-12 was selected because it is a validated, brief measure of health-related QOL and imposes a relatively low respondent burden. This consideration was particularly relevant given the ongoing armed conflict and the associated safety concerns and psychological burden experienced by participants. A shorter instrument was therefore deemed suitable to reduce the time needed for data collection while still relying on an established tool for assessing concurrent validity. The 26-item WHOQOL-BREF was not chosen because its longer format would have placed a heavier burden on respondents in the setting of ongoing wartime data collection.

**Structural (factorial analysis) validity:** EFA was conducted to examine the underlying factor structure of the 35-item AQOL-War and to determine whether the items could be adequately represented by a smaller number of latent factors [29]. Before conducting EFA, we confirmed that the data were suitable for factor analysis using the Kaiser-Meyer-Olkin (KMO) measure of sampling adequacy and Bartlett’s test of sphericity [30]. The KMO values range from 0 to 1. A KMO value of 0.6 or higher is generally considered acceptable, with higher values indicating better suitability for factor analysis. Bartlett’s test of sphericity was used to determine whether the correlation matrix differed significantly from an identity matrix. A statistically significant result (*p* < 0.05) indicated that the correlations among the items were sufficient to support factor analysis. The Kaiser criterion, which retains factors with eigenvalues > 1, was used to determine the number of factors to include. Principal axis factoring (PAF) with varimax rotation was used to identify orthogonal structures in the data. Varimax rotation was applied to facilitate the interpretation of the factor structure and to ensure that items loaded clearly on the appropriate factors. A parallel analysis was utilized to identify the optimal number of factors for extraction. To aid interpretation and avoid overlap between factors, loadings > 0.30 were retained, and cross-loadings were assessed to ensure that each item loaded primarily on only one factor [31].

CFA was conducted to evaluate the hypothesized factor structure of the AQOL-War and assess the extent to which the observed data supported the proposed three-factor model [32]. The CFA was estimated using the maximum likelihood method. Model fit was evaluated using multiple complementary fit indices, including the root mean square residual (RMR), with a desired value of ≤ 0.08, and the root mean square error of approximation (RMSEA), with a satisfactory value of less than 0.08. Also, we estimated fit indices, including the comparative fit index (CFI), with a desired value > 0.9; the goodness of fit index (GFI), with a desired value > 0.9; the Bentler-Bonett non-normed fit index (NNFI); and the Tucker-Lewis Index (TLI), which should exceed 0.9 [33]. The normed ꭓ2 was also used to test the fit of the CFA model. The satisfactory value for normed ꭓ2 was < 5 [34]. The chi-square statistic was interpreted in conjunction with the other fit indices because it is sensitive to sample size.

### Measurement invariance testing

Measurement invariance across sex, place of residence, and employment status was examined using multigroup CFA (MG-CFA) with a sequential hierarchical approach. The analyses examined configural, metric, scalar, strict, and structural invariance by progressively imposing equality constraints across groups. Configural invariance examined whether the same factor structure held across groups; metric invariance evaluated whether factor loadings were equivalent; scalar invariance assessed the equivalence of item intercepts; and strict invariance tested whether residual variances were equal. Model fit at each level of invariance was evaluated using chi-square (χ^2^), CFI, TLI, and RMSEA. Changes in CFI (ΔCFI) and RMSEA (ΔRMSEA) were used to assess differences between nested models. A change in CFI (ΔCFI) of ≤0.01 was considered indicative of invariance, with changes in RMSEA also considered when evaluating model equivalence across groups.

**Known-groups validity**: It was assessed by comparing the AQOL-War total scores across subgroups hypothesized a priori to differ in QOL based on theoretically and empirically relevant characteristics of wartime exposure. Three hypotheses were tested: (1) family loss due to war would be associated with lower QOL; (2) employed participants would report higher QOL than unemployed participants; and (3) participants living in their own homes would report higher QOL scores than those living in emergency shelters or displacement camps. Group comparisons were performed using an independent samples t-tests (family loss, employment status) and one-way analysis of variance (ANOVA) (residence type), with statistical significance set at p < 0.05.

### Ethical considerations

Ethical approval was obtained from the Ethics Committee of the High Institute of Public Health, Alexandria University, Egypt (IRB number: 00013692). Administrative permission to conduct the research was granted by the Sudanese Ministry of Health and Social Development (Humanitarian Aid Commission). The study adhered to international research ethics guidelines. Informed consent was obtained from all participants after explaining the research objectives and benefits. Participants were informed of their right to decline participation or withdraw from the study at any time without consequence. No personal identifying data were obtained, and suitable procedures were implemented to safeguard participants’ anonymity, confidentiality, and privacy during both data collection and analysis.

### Statistical analysis

Data coding and analysis were performed using IBM SPSS Statistics, version 26 (IBM Corp., Armonk, NY, USA). Descriptive statistics were used to summarize participants’ characteristics and AQOL-War scores. Normality was assessed using Q-Q plots and the Kolmogorov-Smirnov test. Normally distributed quantitative variables were summarized with mean and standard deviation (SD), whereas categorical variables were presented as frequencies and percentages. AQOL-War scores were calculated for the total scale and for each of the three domains: environment and security, psychological health, and physical health. The participants’ scores varied from 31 to 143, with a median score of 88, serving as the cutoff point to distinguish between lower and higher QOL. The median score was used as a descriptive reference point to classify participants into relatively lower- and higher-scoring groups for analyses in which this categorization was required. EFA was performed inJASP (Version 0.19), and CFA in JASP and the lavaan R package, to evaluate the measurement model validity [35,36]. Invariance testing across gender, place of residence, and employment status was conducted using multigroup CFA with sequential constraints (configural → metric → scalar → strict → structural), evaluating fit via CFI, RMSEA, and ∆CFI.

## Results

### Characteristics of the participants

The study recruited 635 participants; their mean age was 27.9 ± 9.1 years, 72.1% of them were females, two-thirds (65.4%) lived in urban areas, 67.1% lived in owned residences, 71.5% were single, 70.1% had a university education, and 71.7% were not currently employed. Regarding the current or previous occupation, 47.7% had professional jobs, and 32.8% worked from home. The respondents were from several states across Sudan, with the largest proportion coming from Khartoum State (19.2%). Sixty percent of respondents lived together with their whole family, while 40% were separated from family members because of the war. Approximately one-third (36.2%) lived in households with four to six members, and 27.2% lived with ten or more. Additionally, 21.3% of the respondents reported having lost a family member during the recent war (Table 1).

**Table 1:** Socio-demographic characteristics of Sudanese respondents (N = 635).

Variables	Categories	Frequency (%)
Age	Mean ± SD	27.9 ± 9.1
Gender	Male Female	177 (27.9) 458 (72.1)
Place of residence	Rural/village Urban/governorate	220 (34.6) 415 (65.4)
State of residence	River Nile State Khartoum State White Nile State Red Sea State Gadarif State Kassala State Northern State Gezira State Others	121 (19.1) 122 (19.2) 31 (4.9) 46 (7.2) 28 (4.4) 50 (7.9) 110 (17.3) 107 (16.9) 20 (3.1)
Type of residence	Own house Emergency shelter Displacement camp Others	426 (67.1) 15 (2.3) 29 (4.6) 165 (26.0)
Marital status	Single Married Widowed/divorced	454 (71.5) 163 (25.7) 18 (2.8)
Education level	Less than secondary Secondary / technical University Post-graduate	11 (1.7) 93 (14.7) 445 (70.1) 86 (13.5)
Employment status	Working Not working	180 (28.3) 455 (71.7)
Occupation (previous or current)	Professional job Laborer/ skilled Self-employed/own business Working from home	303 (47.7) 39 (6.1) 85 (13.4) 208 (32.8)
Whole family lives together or separated	Separated Together	254 (40.0) 381 (60.0)
Number of people living together in your household	1–3 4–6 7–9 10+	58 (9.1) 230 (36.2) 174 (27.4) 173 (27.3)
Lost a family member during the war	Yes No	135 (21.3) 500 (78.7)

### Exploratory factor analysis

The KMO value was high (0.889), indicating meritorious sampling adequacy for factor analysis. Bartlett’s test was significant (χ^2^ = 4965.76, *p* < 0.001), indicating that the correlation matrix differed significantly from an identity matrix and that the data were suitable for factor analysis. EFA initially identified three factors with eigenvalues greater than 1 (9.22, 2.95, and 2.45). The three factors explained approximately 41.8% of the total variance. The scree plot and parallel analysis, as indicated in Fig 2, suggested that four factors were sufficient to represent all the items included in the analysis. However, there was a marked decline in eigenvalues after the third factor, and the fourth factor was not considered sufficiently interpretable. Therefore, the three-factor solution was retained because it provided a more parsimonious and conceptually meaningful structure, with most items loading strongly onto the three retained factors.

**Fig 2:**
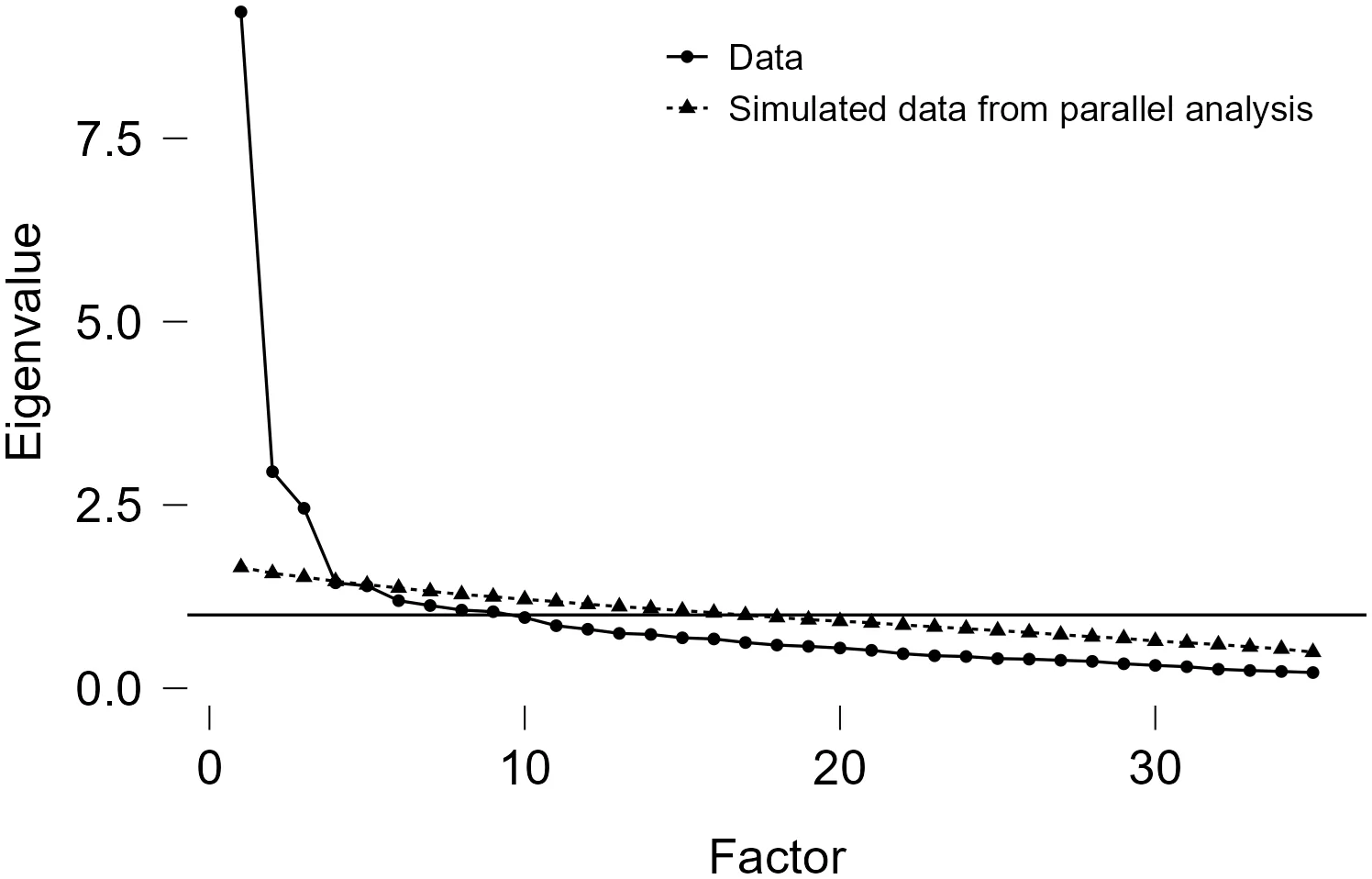
Scree plot of eigenvalues from exploratory factor analysis.

The EFA supported a three-factor structure. Based on the factor-loading pattern and conceptual interpretation of the items, the environment and security domains were merged into one domain, and the original health domain was divided into two domains, psychological health and physical health. Two items were removed from the environment and security domain (E5: Were you displaced from your residence during the current war? and S2: Were you or your family subjected to aggressive/violent behavior?), and two items were removed from the psychological health domain (H9: To what extent do you rate your intimate relationship satisfaction? and H15: How often do you think of suicide during the day?), resulting in a final AQOL-War scale with 31 items: 16 for the environment and security (14 items for environment and 2 items for security), 10 items for psychological health, and 5 items for physical health (S1 File). Loadings of the items ranged between 0.416 and 0.756, as indicated in Table 2.

**Table 2:** Standardized factor loadings of the different items of the AQOL-War.

Item During the war;	Factor 1 Environment and security	Factor 2 Psychological health	Factor 3 Physical health
E1: What is the degree of your satisfaction about your life during current war?	0.487		
E2: What is the degree of your satisfaction with the humanitarian assistance you receive?	0.431		
E3: Does your household have enough money to meet both yours, and your family’s essential needs?	0.416		
E4: To what extent are you financially able to access medical treatment?	0.446		
E6: To what extent are you satisfied with the access to your basic daily needs (food/water/medicine/clothes) in your living area?	0.684		
E7: To what extent are you satisfied with the availability and accessibility of drinking water sources?	0.655		
E8: To what extent are you satisfied with lights (illumination) at night in your current neighborhood/residential area?	0.687		
E9: To what extent are you satisfied with the availability of transportation in your living place (even for emergencies)?	0.756		
E10: To what extent are you satisfied with the accessibility to work?	0.638		
E11: To what extent are you satisfied with the availability and accessibility of schools for children?	0.485		
E12: To what extent is your surrounding environment polluted?	0.513		
E13: To what extent are you satisfied with the availability and accessibility to the source of energy?	0.701		
E14: To what extent are you satisfied with the availability and accessibility of communication facilities and services?	0.663		
E15: To what extent are you satisfied with the availability and accessibility to medical facilities / shelters?	0.679		
S1: To what extent do you feel the area where you live is insecure for you and your family?	0.469		
S3: Are there places in your area that women fear or avoid because they are unsafe?	0.437		
H1: To what extent is your general health affected?		0.504	
H4: To what extent does the war affect your sleep?		0.558	
H8: To what extent do you feel satisfied with your mental health?		0.611	
H10: To what extent do you feel sad during the day?		0.614	
H11: How often do you have a sense of fear about yourself during your daily life?		0.641	
H12: How often do you have a sense of fear about your family members during your daily life?		0.506	
H13: To what extent do you rate the degree of aggressiveness and violence in your behavior?		0.523	
H14: To what extent do you rate your ability to cope with the current situation?		0.562	
H16: To what extent did the war affect your relationship with your family?		0.534	
H17: To what extent has the war negatively affected your social relationships?		0.462	
H2: To what extent has the war caused an injury to you?			0.750
H3: To what extent has the war caused a disability for you?			0.710
H5: To what extent has the war worsened your medical comorbidities or your chronic illness?			0.483
H6: To what extent has the war caused injury to your family members?			0.590
H7: To what extent has the war caused disability for your family members?			0.661

### Confirmatory factor analysis

The CR values indicated strong internal consistency and reliability for the three latent variables. The environment and security domain had a CR of 0.94, the psychological health domain had a CR of 0.83, and the physical health domain had a CR of 0.81, suggesting good reliability for all three domains. The factor model has a much lower chi-square value (χ^2^ = 831.36) than the baseline model (χ^2^ = 5492.23; p < 0.001). Key model fit indices confirmed the quality of the CFA model where RMSEA = 0.06 with 95% CI: [0.054–0.066] indicating reasonable approximate fit. The values of the other model fit indices were RMR = 0.04, CFI = 0.916, GFI = 0.936, TLI = 0.905, and NNFI = 0.905. Factor loadings from CFA showed the strength of the relationship between each observed variable and its corresponding latent factor, with values ranging from 0.317 to 0.853. The AVE was 0.51 for the environment and security domain, 0.33 for psychological health, and 0.45 for physical health. Thus, the environment and security domain met the conventional criterion for convergent validity (AVE ≥ 0.50), whereas the psychological health and physical health domains did not; however, CR values exceeded 0.80 for all domains. The square root of the AVE for each factor was greater than the inter-correlation between factors, confirming discriminant validity (**Table 3**).

**Table 3:** Results of the CFA of AQOL-War. Convergent validity, discriminant validity, and reliability assessment of CFA final model with three latent factors and model fit indices.

Factor	Composite reliability (CR)	Average variance extracted (AVE)	Correlations among latent variables		
			Environment and security	Psychological health	Physical health
**Environment and security**	0.94	0.514	0.717a		
**Psychological health**	0.83	0.331	0.520	0.575a	
**Physical health**	0.81	0.450	0.369	0.249	0.671a
**Model fit indices**	**Normed χ** ^**2**^	**GFI**	**CFI**	**RMSEA**	**RMR**
	2.028	0.936	0.916	0.060	0.040
	**TLI**	**NNFI**			
	0.905	0.905			

Note: GFI: goodness of fit index, CFI: comparative fit index, RMSEA: root mean square error of approximation, RMR: root mean square residual, TLI: Tucker-Lewis Index, NNFI: Non-normed fit index. a) Diagonal elements are the square root of AVE.

A moderate positive relationship (0.52) existed between the environment and security domain and the psychological health domain. A weaker positive relationship (0.369) was observed between the environment and security domain and the physical health domain. The correlation between psychological health and physical health was also weakly positive (0.249). Together with the Fornell–Larcker criterion, these results supported the discriminant validity of the AQOL-War.

Fig 3 demonstrates that item loadings for the environment and security domain ranged from 0.34 (E3) to 0.85 (E9). Similarly, psychological health factor loadings ranged from 0.32 (H1) to 0.79 (H11), and physical health item loadings ranged from 0.64 (H3) to 0.74 (H7). Most items demonstrated moderate to strong loadings on their respective factors, supporting the adequacy of the proposed three-factor measurement structure. This factor structure was further supported by the overall CFA model-fit indices.

**Fig 3:**
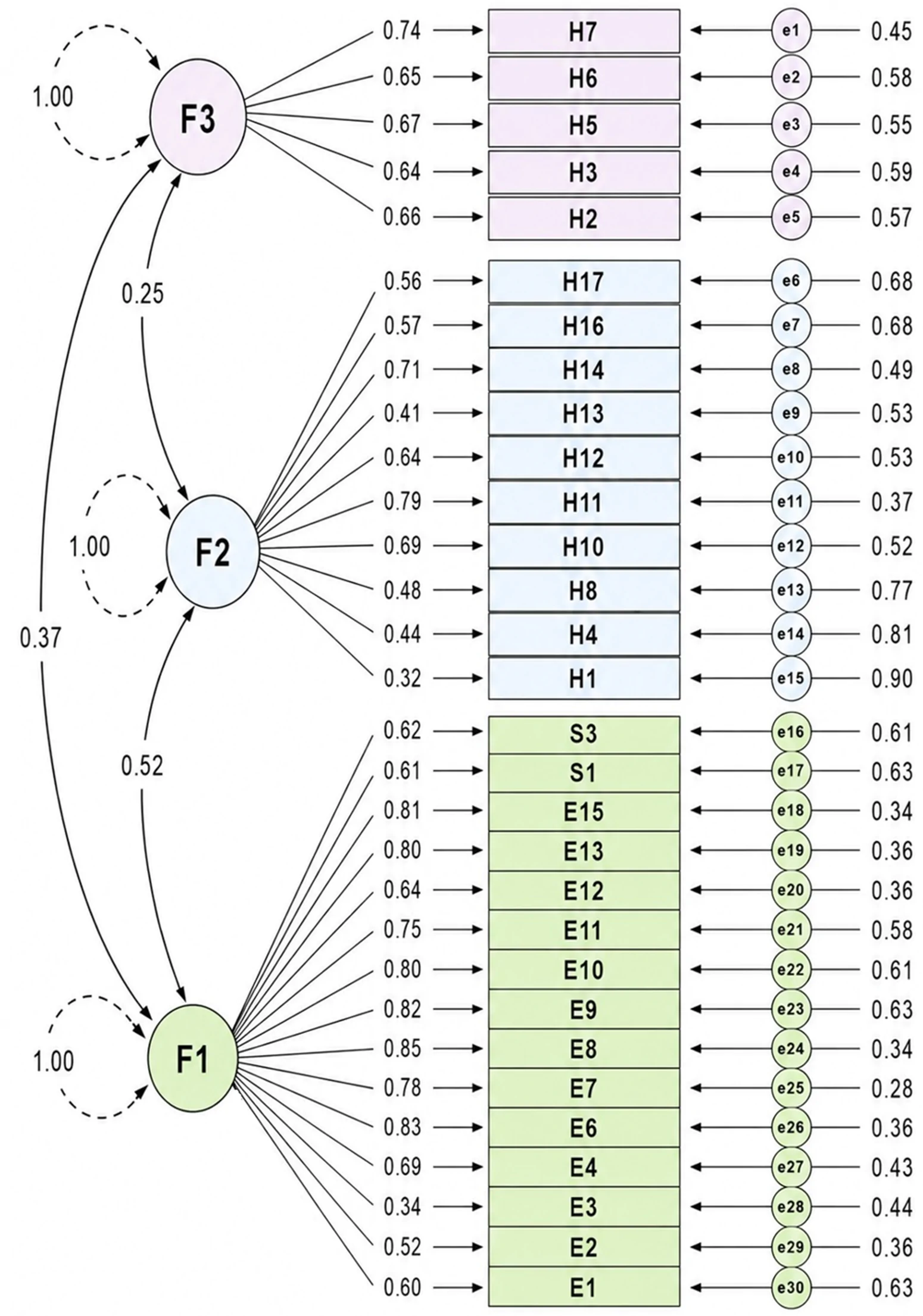
Confirmatory factor analysis of the AQOL-War.

### The invariance testing results

The measurement invariance results showed varying levels of support for the AQOL-War across gender, place of residence, and employment status. Across gender, the configural model showed RMSEA = 0.072, while the CFI was 0.881, which was below the conventional threshold of 0.90. Nevertheless, subsequent constraints from the metric through structural models produced only minimal changes in model fit. The changes in CFI remained within the prespecified criterion (|ΔCFI| ≤ 0.01), ranging from 0.000 to −0.006, while changes in RMSEA were negligible. These findings indicate that the measurement structure remained relatively stable across male and female participants, providing support for measurement invariance across gender groups.

For the place of residence, the configural model yielded CFI = 0.875 and RMSEA = 0.075. Although the CFI was below the conventional 0.90 threshold, the metric and scalar models showed changes in CFI within the prespecified criterion (ΔCFI = −0.005 and −0.010, respectively), accompanied by only minor changes in RMSEA (0.000 and 0.002, respectively). These findings support metric and scalar invariance across place-of-residence groups. However, the strict invariance model resulted in ΔCFI = −0.013, exceeding the prespecified criterion of |ΔCFI| ≤ 0.01, although the corresponding change in RMSEA remained small (ΔRMSEA = 0.001). Therefore, strict invariance was not supported based on the ΔCFI criterion.

For employment status, the configural model yielded CFI = 0.893 and RMSEA = 0.069. The metric and scalar models demonstrated only small changes in model fit, with ΔCFI values of −0.004 and −0.007, respectively, and corresponding ΔRMSEA values of 0.000 and 0.001. These results support metric and scalar invariance across employment-status groups. In contrast, the strict invariance model produced ΔCFI = −0.011, slightly exceeding the prespecified criterion of |ΔCFI| ≤ 0.01, despite a small change in RMSEA (ΔRMSEA = 0.002). Thus, strict invariance was not fully supported according to the ΔCFI criterion. The subsequent structural model showed only a small additional change in fit (ΔCFI = −0.003; ΔRMSEA = 0.000). Overall, the results provide support for measurement invariance up to the scalar level across employment-status groups, while strict invariance should be interpreted with caution (Table 4).

**Table 4:** Measurement invariance of the AQOL-War Scale across gender, place of residence, and employment status.

Model	χ²	df	CFI	TLI	RMSEA	ΔCFI	ΔRMSEA
**Gender**							
Configural	1426.795	820	0.881	0.866	0.072	–	–
Metric	1458.676	848	0.881	0.869	0.071	0.000	−0.001
Scalar	1505.591	876	0.877	0.869	0.071	−0.004	0.000
Strict	1589.447	928	0.871	0.870	0.071	−0.006	0.000
Structural	1614.079	937	0.868	0.869	0.071	−0.003	0.000
**Place of residence**							
Configural	1479.004	820	0.875	0.858	0.075	–	–
Metric	1534.058	848	0.870	0.857	0.075	−0.005	0.000
Scalar	1617.115	876	0.860	0.851	0.077	−0.010	0.002
Strict	1736.567	928	0.847	0.846	0.078	−0.013	0.001
Structural	1769.257	937	0.842	0.844	0.079	−0.005	0.001
**Employment status**							
Configural	1379.968	820	0.893	0.879	0.069	–	–
Metric	1427.267	848	0.889	0.879	0.069	−0.004	0.000
Scalar	1494.353	876	0.882	0.875	0.070	−0.007	0.001
Strict	1605.293	928	0.871	0.870	0.072	−0.011	0.002
Structural	1628.964	937	0.868	0.869	0.072	−0.003	0.000

Note: χ^2^: chi squared statistic, df: degrees of freedom**,** CFI: comparative fit index, TLI: Tucker-Lewis Index, RMSEA: root mean square error of approximation.

### Reliability and Item-level analysis

Table 5 shows the reliability analysis and convergent validity of the AQOL-War. The overall bach’s alpha for the QOL tool was 0.93. Cronbach’s alpha values for each of the domains “environment and security”, “psychological health”, and “physical health” were 0.94, 0.84, and 0.83, respectively, indicating good internal consistency.

**Table 5:** Descriptive statistics, internal consistency, convergent validity, and discriminant validity of the AQOL-War scale.

Domain / Items	Mean ± SD	Item–total correlation	Cronbach’s α	Split-half reliability	Discriminant validity (factor correlations)
Environment and security domain			0.94	SB = 0.92; Guttman = 0.92	Environment and security domain vs psychological health domain = 0.572**; Environment and security vs physical health = 0.288**
E1	2.76 ± 1.46	0.531**			
E2	2.21 ± 1.29	0.403**			
E3	3.09 ± 1.18	0.485**			
E4	3.57 ± 1.13	0.515**			
E6	3.22 ± 1.24	0.688**			
E7	3.23 ± 1.24	0.604**			
E8	3.02 ± 1.38	0.652**			
E9	2.86 ± 1.27	0.711**			
E10	2.82 ± 1.24	0.627**			
E11	2.29 ± 1.26	0.519**			
E12	2.52 ± 1.07	0.549**			
E13	2.61 ± 1.18	0.661**			
E14	2.77 ± 1.26	0.650**			
E15	2.81 ± 1.18	0.649**			
S1	3.39 ± 1.41	0.522**			
S3	3.39 ± 1.43	0.489**			
Psychological health domain			0.84	SB = 0.78; Guttman = 0.78	Psychological health domain vs Physical health domain = 0.153**
H1	3.03 ± 1.31	0.447**			
H4	2.79 ± 1.39	0.519**			
H8	2.60 ± 1.34	0.618**			
H10	2.45 ± 1.25	0.615**			
H11	2.59 ± 1.42	0.633**			
H12	2.05 ± 1.28	0.518**			
H13	3.22 ± 1.44	0.505**			
H14	2.62 ± 1.31	0.547**			
H16	3.31 ± 1.52	0.550**			
H17	2.67 ± 1.44	0.459**			
Physical health domain			0.83	SB = 0.79; Guttman = 0.77	Physical health domain vs psychological health domain = 0.153**
H2	4.47 ± 1.18	0.611**			
H3	4.71 ± 0.99	0.587**			
H5	3.73 ± 1.57	0.407**			
H6	4.03 ± 1.51	0.532**			
H7	4.49 ± 1.19	0.532**			

^1^SB, Spearman–Brown coefficient; ** *p* <0.01.

Concerning the environment and security domain, the items (E1-S2) showed varying mean scores, ranging from 2.21 ± 1.29 to 3.57 ± 1.13. The Spearman-Brown coefficient and the Güttman Split-Half coefficient were reported as 0.92. Regarding the psychological health domain, the items showed mean scores ranging from 2.05 ± 1.28 to 3.31 ± 1.52. The Spearman-Brown coefficient and the Güttman Split-Half coefficient were equal to 0.78. Regarding the physical health domain, the items showed mean scores ranging from 3.73 ± 1.57 to 4.71 ± 0.99. The Spearman-Brown coefficient equals 0.79, and the Güttman Split-Half coefficient equals 0.77.

All items showed positive item-total correlations with their respective domain scores, indicating consistency of items with the overall scale and good convergent validity. Tables A2–A4 in S3 File and Figs A1–A3 in S2 File provide the inter-item correlation matrices for the three factors. Correlation coefficients were significant (p < 0.01), indicating that the items included in each factor were highly correlated and measured the same construct.

### Concurrent validity

There was a moderate positive correlation between the AQOL-War (Version I) score and the Arabic version of the SF-12 score (*r* = 0.675, p < 0.001), providing evidence of concurrent validity (Fig A4 in S2 File). The positive correlation indicates that higher QOL scores on the AQOL-War were associated with higher health-related QOL scores on the SF-12, while the correlation was not so high as to suggest that the two instruments measure identical constructs.

### Known-groups validity testing

Participants who reported loss of a family member during the war had significantly lower QOL scores (mean = 75.99 ± 26.47) compared to those who did not report such loss (mean = 91.24 ± 23.11, p < 0.001). Employed participants had significantly higher AQOL-War scores (mean = 92.54 ± 20.82) compared with unemployed participants (mean = 85.00 ± 26.11, p = 0.024). Statistically significant differences in AQOL-War scores were also observed across residence types (p < 0.001). Participants living in their own homes reported the highest QOL scores (mean = 92.25 ± 24.17), followed by those living in emergency shelters (mean =82.67 ± 32.54) and other types of residences (mean =81.46 ± 23.06), while the lowest scores were observed among those living in displacement camps (mean = 62.07 ± 22.00).

### Cut-off points and QOL prevalence

QOL scores, evaluated using median cut-off points, revealed that total AQOL-War scores ranged from 31 to 143 (median = 88), with 51.9% of participants reporting poor overall QOL and 48.1% reporting good QOL. Within specific subdomains, scores ranged from 16 to 77 for environment and security (median = 44; 50.2% poor QOL), 10–50 for psychological health (median = 24; 53.3% poor QOL), and 5–25 for physical health (median = 22; 54.7% poor QOL) (Fig 4).

**Fig 4:**
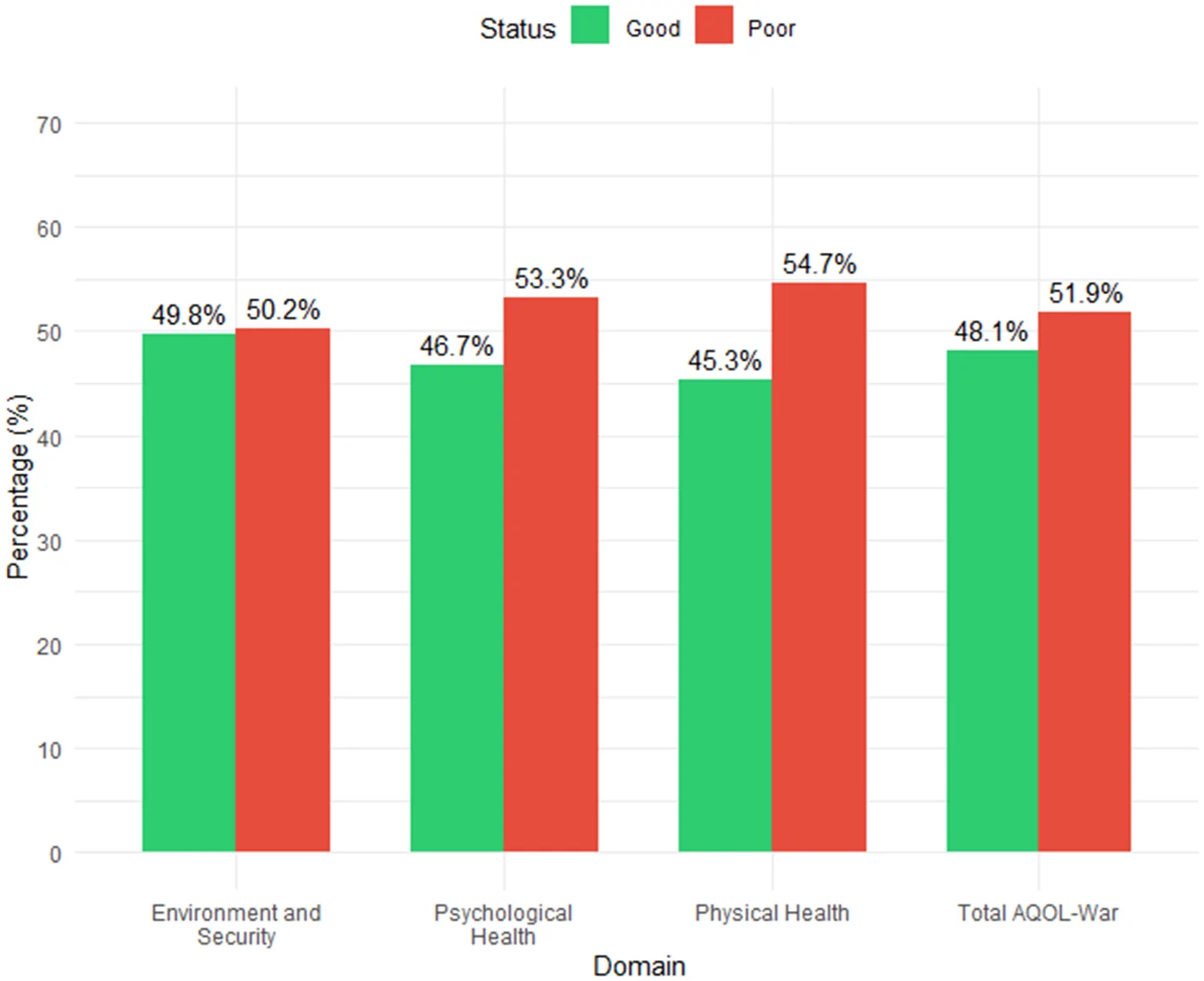
Prevalence of good and poor quality of life overall and across subdomains based on median cut-off points.

## Discussion

To the best of the authors’ knowledge, the AQOL-War is among the first tools specifically conceptualized to capture the transformation of QOL into a survival-oriented construct during war and to be psychometrically evaluated for use in an Arabic-speaking, war-affected population. The final AQOL-War comprised 31 items across three factors: environment and security (16 items), psychological health (10 items), and physical health (5 items). This three-factor structure reflects dimensions of QOL that are particularly relevant to the conditions of war, extending beyond conventional QOL frameworks developed primarily in non-war settings. The current validation strategy addressed most key measurement properties outlined in the COSMIN (Consensus-based standards for the selection of health Measurement Instruments) framework, including content validity, structural validity, internal consistency, and hypothesis testing for construct validity. Measurement invariance was also examined across gender, place of residence, and employment status using MG-CFA, providing evidence that the instrument measures the underlying constructs comparably across these groups [37].

The most theoretically important finding is the empirical convergence of the originally hypothesized environment and security domains into a single highly cohesive Factor (Cronbach’s α = 0.94; AVE = 0.514; CR = 0.94). This merging of dimensions into a single construct highlights how a war environment tends to reshape QOL perceptions, extending research on QOL measurements in war-affected populations [16,22,38,39]. Unlike peacetime QOL frameworks such as the WHOQOL-BREF [12], where environment and security are treated as separate dimensions, our results showed that in war settings these concepts converge into a single construct. Items related to drinking water (E6), energy (E12), emergency transport (E8), night-time illumination (E7), and neighborhood safety (S1, S2) loaded onto the same factor, reflecting how environmental conditions associate with survival and security during war. These findings align with studies of war-affected populations showing that conventional QOL structures require modification in war settings [14,16,40]. A recent assessment of WHOQOL-BREF in Colombian war survivors found that its four-factor structure failed to fit war-affected data and required dimensionality re-specification [14]. In the present study, the psychological health (Cronbach’s α = 0.84) and physical health (Cronbach’s α = 0.83) domains demonstrated good internal consistency. Together with the emergence of a combined environment and security factor, these findings suggest that QOL in war-affected populations may be structured around interconnected environmental, security, physical, and psychological dimensions that differ from conventional peacetime QOL models.

Four items were excluded during EFA because of their low factor loadings: forced displacement, exposure to violence, intimate-relationship satisfaction, and suicidal ideation. These exclusions were considered theoretically and statistically meaningful. Forced displacement and violence exposure are salient characteristics of war, but their relatively low factor loadings suggest that they may function more as indicators of exposure to war-related events than as reflective indicators of the underlying QOL construct [41]. Their limited contribution to the latent factor structure may therefore reflect differences in how participants experienced or interpreted these events. Similarly, intimate-relationship satisfaction and suicidal ideation may represent more specific aspects of psychosocial experience that are not sufficiently aligned with the broader psychological health construct measured by the AQOL-War, a pattern previously observed in a Palestinian study [40]. The low loading of the suicidal ideation item may also be due to a strongly skewed response pattern, possibly involving a floor effect, as well as underreporting driven by social desirability and by cultural or religious norms that discourage disclosure of suicidal thoughts [42].

The final three-factor model explained 41.8 % of total variance. Although this is below the conventional recommendations of 50–60% [31,43], the proportion of explained variance should be interpreted in the context of the study population and the complexity of the construct rather than as a standalone criterion. Methodological guidance recommends considering explained variance alongside theoretical coherence, factor interpretability and stability, item loadings, model fit, and reliability [44–46]. In the present study, the three-factor solution provided a theoretically coherent and interpretable structure, with satisfactory internal consistency and acceptable CFA model fit. Although parallel analysis suggested the potential presence of a fourth factor, this factor was not sufficiently interpretable or conceptually distinct; therefore, the more parsimonious three-factor solution was retained. In health and social science research on highly stressed populations, lower explained variance is common due to the complexity of their real-world lived experience. The final CFA model demonstrated satisfactory fit indices, including a normed χ^2^ of less than five [34,47], providing further support for the retained factor structure.

The AVE values for psychological health (0.33) and physical health (0.45) fell below the classical 0.50 threshold for convergent validity. However, both domains demonstrated satisfactory CR (0.83 and 0.81, respectively), indicating good internal consistency despite the relatively high proportion of item-level error variance. Fornell and Larcker [48] emphasized that AVE should be considered alongside composite reliability when evaluating convergent validity. In the present study, the high CR values provide supportive evidence for the reliability of these constructs, although the AVE results indicate that convergent validity was not fully established according to the conventional criterion. The environment and security domain met both conventional criteria, with an AVE of 0.514 and CR of 0.942. Assessment of common-method bias using Harman’s single-factor test showed that the first unrotated factor explained only 26% of variance, well below the conventional 50% threshold of concern [49]. This finding provides no indication of substantial common-method bias based on this diagnostic; however, Harman’s single-factor test has recognized limitations and should not be considered definitive evidence that common-method bias was absent.

It is worth noting that existing peacetime QOL instruments often fail to capture the realities of active war contexts. Revalidation studies from Colombia and Palestine showed that tools such as the WHOQOL-BREF required major structural modifications to fit war-affected populations and struggled to address insecurity and political violence adequately [14,16,40]. Among war survivors in Palestine, the original four-factor model failed to reach acceptable fit and required bifactor re-specification [16]. Another Palestinian study eliminated the social domain and added a *human-insecurity* sub-instrument to capture political-violence dimensions that the WHOQOL-BREF could not accommodate [40]. Other instruments, including the MANSA [17], Self-Reliance Index [50], SF-12 [28], and GHQ-12 [51], measure selected aspects of well-being or health status, but do not comprehensively assess the combined environmental, security, psychological and physical dimensions relevant to a war context. MANSA emphasizes satisfaction with relational, occupational, and leisure life, the domains most disrupted by mass displacement, but achieves a modest internal consistency (α = 0.74) [17]. The Self-Reliance Index, developed for refugee and internally displaced populations, incorporates housing, food, safety, and healthcare, but is a household-level resilience measure rather than an individual QOL instrument and ignores the psychological and physical dimensions [50]. The SF-12 and GHQ-12 perform well in war-affected populations [28,51], but they are health-status screeners rather than QOL tools and were never intended to capture environmental safety. On the other hand, the developed AQOL-War addressed these gaps by using a war-related item pool rather than modifying peacetime frameworks, resulting in a multidimensional instrument integrating environmental safety with psychological and physical health. Its strong internal consistency (overall α = 0.931) further supports its reliability.

The SF-12 was selected for concurrent criterion validity because it was brief, Arabic-validated, and feasible under unstable field conditions where long interviews increased respondent burden and security risks [28]. The moderate correlation between the AQOL-War and the SF-12 (r = 0.675, p < 0.001) indicated meaningful convergence on their shared health constructs (physical and mental health) while differing systematically on the war-specific dimensions absent from SF-12. The magnitude of the correlation is consistent with the AQOL-War and SF-12 assessing related but non-identical constructs [27]. This value falls within the COSMIN-recommended range of 0.50–0.80 for instruments [37]. It is large enough to demonstrate meaningful convergent alignment with an established health-status measure, yet small enough to confirm that the AQOL-War captures QOL dimensions not fully represented by the SF-12 [27]. These findings therefore support the interpretation that the AQOL-War complements rather than duplicates existing generic health-status instruments. Although the correlation for the AQOL-War scale is satisfactory, it is lower than that reported for the MANSA questionnaire where the correlation between it and the previously validated Lancashire Quality of Life Profile (LQLP) was 0.83 [17]. Future studies should compare and extend concurrent testing with additional Arabic-validated QOL and trauma-specific tools.

The AQOL-War MG-CFAs demonstrated configural and metric invariance across gender, residence, and employment groups, indicating that the instrument organizes items into the same factor structure with comparable loadings across subgroups and can therefore support comparative monitoring of QOL without major measurement bias [52]. This structural equivalence is complemented by preliminary known-groups (construct) validity within the dataset. AQOL-War scores showed a marked gradient across groups differing a priori in vulnerability, with the lowest scores among displacement-camp residents and the war-bereaved (21.3% of respondents). This pattern parallels the dose–response relationship between war intensity and lower QOL reported in other war settings [22] and confirms that the instrument discriminates between populations with established differences in exposure and living conditions.

Beyond the AQOL-War’s psychometric performance, the study findings reflect the severe humanitarian impact of the Sudanese war. More than half of respondents had poor overall QOL (51.9%), with the physical health domain being the most impacted (54.7%). This compromised QOL resulted from restricted access to basic needs, humanitarian aid, schooling, and emergency transportation, driven by the documented collapse of essential infrastructure [7,41]. Reporting of family loss and family separation further aligned with independent estimates of mortality and displacement from the same war [6,53,54]. These findings are consistent with evidence from other war settings, including Colombia [22], Ukraine [39,55] and Palestine [56] showing that reduced QOL is strongly linked to war-related political violence, insecurity, and disrupted living conditions. The AQOL-War scale therefore reflects a broader war-related pattern where environmental safety forms the foundation of psychological and physical well-being. Thus, the AQOL-War instrument may have potential for longitudinal monitoring, but responsiveness and temporal stability require evaluation in longitudinal studies. Its short administration time (approximately 15 minutes) makes it practical for rapid needs assessment and household surveys in Arabic-speaking war settings, including Sudan, Yemen, Syria, Palestine, Lebanon, and Iraq.

**Implications of the study**: The AQOL-War scale may help identify individuals in need of further psychosocial and material-needs assessment. Low psychological-health domain scores, particularly when paired with items capturing sustained sadness, fear for family, and inability to cope, identify candidates for mental and psychosocial support, while very low security-item scores should trigger referral to protection actors, especially for female respondents in regions with documented gender-based violence. Nevertheless, the AQOL-War is not a clinical diagnostic tool and should be used alongside established symptom-specific tools and clinical assessment where possible.

The developed AQOL-War scale can offer a supportive framework for evidence-based humanitarian action. For example, in an operational context, a localized and significant drop in the security domain score, detected through periodic assessments, could potentially inform humanitarian monitoring and prioritization, pending validation of clinically and operationally meaningful thresholds. Specifically, if a district exhibits a sharp decline in security-related QOL, humanitarian organizations could prioritize that area for immediate resource reallocation, such as deploying mobile health clinics or increasing distributions of emergency supplies to mitigate the heightened environmental and physical risks. By integrating these quantitative metrics into standard monitoring and evaluation workflows, organizations can move toward more proactive, data-driven strategies that address the most urgent needs of populations in volatile war zones.

### Strengths, limitations, and future directions

A key strength of this study is that the AQOL-War was developed and validated during active war, using combined online and face-to-face recruitment and independent samples for EFA and CFA. However, several limitations should be acknowledged. Convenience and snowball sampling resulted in a sample skewed toward young, female, single, and university-educated respondents. Consequently, the instrument performance in older adults, lower-literacy participants, and residents of the most severely affected regions (Darfur and Kordofan) remains insufficiently examined. Furthermore, concurrent validity was assessed using only a single external instrument. In addition, the cross-sectional nature limits conclusions regarding the instrument’s responsiveness and sensitivity to change over time. Future research should prioritize cross-validation of the AQOL-War in other Arabic-speaking populations affected by war (Gaza, the West Bank, Yemen, Syria, Iraq, and Lebanon). Longitudinal studies are also needed to establish test-retest reliability. Additional priorities include item-response-theory modelling to develop a shorter version for frontline humanitarian use and the development of a complementary instrument capable of capturing collective and intergenerational dimensions of war-related QOL. Despite these limitations, the AQOL-War represents a theoretically grounded, and operationally feasible instrument with promising preliminary psychometric performance for assessing QOL in Arabic-speaking war settings. By empirically demonstrating the convergence of environment and security domains into a single construct, the instrument not only enables more accurate measurement of QOL during war but also provides a framework for examining how war structurally transforms human well-being itself.

## Conclusion

This study provides evidence of structural, discriminant, concurrent, and known-groups validity, partial convergent validity, and good internal consistency. The cross-group measurement invariance of the AQOL-War within the Sudanese war context was satisfactory as well. The 31-item, three-factor structure (environment and security, psychological health, and physical health) captured a survival-based configuration of QOL in which environment and security converge into a single environmental-safety construct. These findings support the theoretical premise that peacetime QOL tools do not transfer unchanged into active war environments. Given that concurrent validity rested on a single external instrument, the sample was recruited by convenience under wartime constraints, and test–retest reliability and responsiveness were not assessed, the AQOL-War should be considered a preliminarily validated rather than fully established instrument. External validation in other Arabic-speaking war-affected populations and longitudinal application to establish temporal stability and responsiveness are the next priorities. Even at this stage, the AQOL-War addresses a measurement gap of immediate operational relevance for the war-affected populations it was designed to serve.

## Supporting information

S1 File Supplementary questionnaire, English version II. English version of the study questionnaire (Version II) used in the validation of the AQOL-War scale.
(DOCX)

S2 File Supplementary figures. Fig A1. Pearson’s r heatmap of items in the Environment and Security domain. Fig A2. Pearson’s r heatmap of items in the Psychological Health domain. Fig A3. Pearson’s r heatmap of items in the Physical Health domain. Fig A4. Correlation between the AQOL-War score and the Arabic version of the 12-item Short-Form Health Survey.
(DOCX)

S3 File Supplementary tables. Table A1. Content Validity Index (CVI) for the studied questionnaire. Table A2. Inter-item correlations and p-values in the Environment and Security domain. Table A3. Inter-item correlations and p-values in the Psychological Health domain. Table A4. Inter-item correlations and p-values in the Physical Health domain.
(DOCX)

S4 File CFA. Dataset used for the confirmatory factor analysis.
(XLSX)

S5 File EFA. Dataset used for the exploratory factor analysis.
(XLSX)

S6 File PLOS Human Participants Research Checklist. Completed PLOS human participants research checklist for this study.
(PDF)
