# Supplementary material for: Development and preliminary validation of the Arabic Quality of Life During War Scale (AQOL-War)
Source: PLoS One. 2026 Oct 29;21(10):e0358784. doi: 10.1371/journal.pone.0358784 (PMC13718929; doi:10.1371/journal.pone.0358784)
Supplement: Supplementary file 2 [file pone.0358784.s001.docx]

**Development and Preliminary Validation of the Arabic Quality of Life During War Scale (AQOL-War)**

**Answer the following questionnaire regarding the current conflict/ war:**

I. **Socio-Demographic Data:**
1. Age:
2. Gender:
   a) Male
   b) Female
3. Place of residence:
   a) Urban/governorate
   b) Rural/village
4. In which governorate are you living in?

…………………………………………

5. Type of residence:
   a) Own house,
   b) Emergency shelter,
   c) Displacement camp,
   d) Other: mention…...
6. Marital status:
   a) Married,
   b) Single,
   c) Divorced,
   d) Widowed.
7. Education
   a) Read and write,
   b) Primary education,
   c) Preparatory (middle) education,
   d) Secondary education/ technical education,
   e) University education,
   f) Post-graduate education.
8. Current occupation
   a) Not working/ housewife,
   b) Working.
9. Current/previous work
   a) Professional job (doctor- teacher – engineer… etc),
   b) Laborer/ skillful,
   c) Self-employed/own business,
   d) Working from home.
10. Number of persons living together in your household:

…………………………………………………………..

11. Whole family lives
    a) Together
    b) Separated
12. Have you lost a family member during the recent war?
    a) Yes
    b) No
II. **Please Answer the Following Questions About the Quality of Life and Sense of Security During the Current War:**

- **Environmental Domain:**

1. What is the degree of your satisfaction about your life during current war?
1. Extremely Dissatisfied,
2. Mostly Dissatisfied,
3. Neutral,
4. Mostly Satisfied,
5. Extremely Satisfied.
2. What is the degree of your satisfaction with the humanitarian assistance you receive?
1. Extremely Dissatisfied,
2. Mostly Dissatisfied,
3. Neutral,
4. Mostly Satisfied,
5. Extremely Satisfied.
3. Does your household have enough money to meet, both yours and your family’s essential needs?
1. No, I have to borrow money,
2. No.
3. Neutral,
4. Yes,
5. Yes, and I can save money.
4. To what extent are you financially able to access medical treatment?
1. Extremely not capable,
2. Not capable,
3. Neutral,
4. Yes, some services,
5. Yes, to all services.

- **Regarding your current neighborhood / living area please answer questions from number 5 to number 14:**

5. To what extent are you satisfied with the access to your basic daily needs (food/water/medicine/clothes) in your living area?
1. Extremely dissatisfied,
2. Dissatisfied,
3. Neutral,
4. Satisfied,
5. Extremely satisfied.
6. To what extent are you satisfied with the availability and accessibility of drinking water sources?
1. Extremely dissatisfied,
2. Dissatisfied,
3. Neutral,
4. Satisfied,
5. Extremely satisfied.
7. To what extent are you satisfied with lights (illumination) at night in your current neighborhood/residential area?
1. Extremely dissatisfied,
2. Dissatisfied,
3. Neutral,
4. Satisfied,
5. Extremely satisfied.
8. To what extent are you satisfied with the availability of transportation in your living place (even for emergencies)?
1. Extremely dissatisfied,
2. Dissatisfied,
3. Neutral,
4. Satisfied,
5. Extremely satisfied.
9. To what extent are you satisfied with the accessibility to work?
1. Extremely dissatisfied,
2. Dissatisfied,
3. Neutral,
4. Satisfied,
5. Extremely Satisfied.
10. To what extent are you satisfied with the availability and accessibility of schools for children?
1. Extremely dissatisfied,
2. Dissatisfied,
3. Neutral,
4. Satisfied,
5. Extremely satisfied.
11. To what extent is your surrounding environment polluted?
1. Extremely polluted,
2. Polluted,
3. Neutral,
4. Clean,
5. Extremely clean.
12. To what extent are you satisfied with the availability and accessibility to the source of energy?
1. Extremely dissatisfied,
2. Dissatisfied,
3. Neutral,
4. Satisfied,
5. Extremely satisfied.
13. To what extent are you satisfied with the availability and accessibility of communication facilities and services?
1. Extremely dissatisfied,
2. Dissatisfied,
3. Neutral,
4. Satisfied,
5. Extremely satisfied.
14. To what extent are you satisfied with the availability and accessibility to medical (facilities / shelters)?
1. Extremely dissatisfied,
2. Dissatisfied,
3. Neutral,
4. Satisfied,
5. Extremely satisfied.

- **Security Domain:**

Security issues: please answer regarding the current conflict/war:

15. To what extent do you feel the area where you live is insecure for you and your family?

Extremely unsafe	1	2	3	4	5	extremely safe

16. There are places in your area that women fear or avoid because they are unsafe:

Extremely unsafe	1	2	3	4	5	extremely safe

III. **Please Answer the Following Questions Regarding Public Health and Mental Health:**

Regarding public and mental health satisfaction during current war (rate your affection)

1. To what extent is your general health affected?

Extremely affected by war	1	2	3	4	5	extremely not affected

2. To what extent does the war affect your sleep?

Extremely affected by war	1	2	3	4	5	extremely not affected

3. To what extent do you feel satisfied with your mental health?

Extremely not satisfied	1	2	3	4	5	extremely satisfied

4. To what extent do you feel sad during the day?

Rarely	1	2	3	4	5	most of the time

5. How often do you have a sense of fear about yourself during your daily life?

Most of the time	1	2	3	4	5	rarely

6. How often do you have a sense of fear about your family members during your daily life?

Most of the time	5	4	3	2	1	rarely

7. To what extent do you rate the degree of aggressiveness and violence in your behavior?

Rarely	1	2	3	4	5	very aggressive and violent

8. To what extent do you rate your ability to cope with the current situation?

Extremely affected	5	4	3	2	1	extremely not affected

9. To what extent did the war affect your relationship with your family members?

Extremely not affected	1	2	3	4	5	extremely affected

10. To what extent has the war negatively affected your social relationships?

Extremely not affected	1	2	3	4	5	extremely affected

IV. **Physical Health Domain:**

Please answer the following questions about your physical health in the

current conflict/war period:

1. To what extent has the war caused an injury to you?

Extremely affected by war	1	2	3	4	5	extremely not affected

2. To what extent has the war caused disability for you?

Extremely affected by war	1	2	3	4	5	extremely not affected

3. To what extent has the war worsened your medical comorbidities or your chronic illness?

Extremely affected by war	1	2	3	4	5	extremely not affected

4. To what extent has the war caused injury to your family members?

Extremely affected by war	1	2	3	4	5	extremely not affected

5. To what extent has the war caused disability for your family members?

Extremely affected by war	1	2	3	4	5	extremely not affected

**Thank You**
