# Supplementary material for: Development and preliminary validation of the Arabic Quality of Life During War Scale (AQOL-War)
Source: PLoS One. 2026 Oct 29;21(10):e0358784. doi: 10.1371/journal.pone.0358784 (PMC13718929; doi:10.1371/journal.pone.0358784)
Supplement: Supplementary file 3 [file pone.0358784.s002.docx]

**Figure (A1): Pearson's r heatmap of items in environment and security domain**

**Figure (A2): Pearson's r heatmap of items in psychological health domain**

**Figure (A3): Pearson's r heatmap of items in physical health domain**

**Figure (A4): correlation between** **the score of AQOL-Conflict and the Arabic version of the “12-item short-form health survey questionnaire**
