# Supplementary material for: Development and preliminary validation of the Arabic Quality of Life During War Scale (AQOL-War)
Source: PLoS One. 2026 Oct 29;21(10):e0358784. doi: 10.1371/journal.pone.0358784 (PMC13718929; doi:10.1371/journal.pone.0358784)
Supplement: Supplementary file 4 [file pone.0358784.s003.docx]

| Domain \| Item \| Number of expert accepted the item (out of 6) \| Item-level CVI (I-CVI) (Number of experts accepted the item/ total number of experts) \| Scale-level CVI (S-CVI) S-CVI/Ave (sum of I-CVI/ total number of items); Environment \| 1 \| 5 \| 0.83 \| 12.81/15= 0.854; \| 2 \| 5 \| 0.83 \|; \| 3 \| 5 \| 0.83 \|; \| 4 \| 5 \| 0.83 \|; \| 5 \| 5 \| 0.83 \|; \| 6 \| 5 \| 0.83 \|; \| 7 \| 4 \| 0.67 \|; \| 8 \| 4 \| 0.67 \|; \| 9 \| 6 \| 1.0 \|; \| 10 \| 5 \| 0.83 \|; \| 11 \| 6 \| 1.0 \|; \| 12 \| 5 \| 0.83 \|; \| 13 \| 5 \| 0.83 \|; \| 14 \| 6 \| 1.0 \|; \| 15 \| 6 \| 1.0 \|; Health \| 1 \| 5 \| 0.83 \| 13.63/17= 0.802; \| 2 \| 5 \| 0.83 \|; \| 3 \| 4 \| 0.67 \|; \| 4 \| 5 \| 0.83 \|; \| 5 \| 5 \| 0.83 \|; \| 6 \| 5 \| 0.83 \|; \| 7 \| 4 \| 0.67 \|; \| 8 \| 5 \| 0.83 \|; \| 9 \| 5 \| 0.83 \|; \| 10 \| 5 \| 0.83 \|; \| 11 \| 5 \| 0.83 \|; \| 12 \| 5 \| 0.83 \|; \| 13 \| 5 \| 0.83 \|; \| 14 \| 4 \| 0.67 \|; \| 15 \| 5 \| 0.83 \|; \| 16 \| 5 \| 0.83 \|; \| 17 \| 5 \| 0.83 \|; Security \| 1 \| 5 \| 0.83 \| 2.33/3=0.777; \| 2 \| 5 \| 0.83 \|; \| 3 \| 4 \| 0.67 \|; Total \| \| \| 28.77 \| 28.77/35=0.822 **Table (A1) for the Content Validity Index (CVI) For the studied questionnaire intended to be validated** **Table (A2): inter item correlations of items and their p-values in environment and security domain** | | | | | | | | | | | | | | | | | | | | | | | | | | | | | | | | | | |  |
| --- | --- | --- | --- | --- | --- | --- | --- | --- | --- | --- | --- | --- | --- | --- | --- | --- | --- | --- | --- | --- | --- | --- | --- | --- | --- | --- | --- | --- | --- | --- | --- | --- | --- | --- | --- |
| **Variable** | | **E1** | | **E2** | | **E3** | | **E4** | | **E6** | | **E7** | | **E8** | | **E9** | | **E10** | | **E11** | | **E12** | | **E13** | | **E14** | | **E15** | | **S1** | | **S3** | | |  |
| **E1** |  |  | — |  |  |  |  |  |  |  |  |  |  |  |  |  |  |  |  |  |  |  |  |  |  |  |  |  |  |  |  |  |  |  | |
|  |  |  | — |  |  |  |  |  |  |  |  |  |  |  |  |  |  |  |  |  |  |  |  |  |  |  |  |  |  |  |  |  |  |  | |
| **E2** |  |  | 0.360 |  | — |  |  |  |  |  |  |  |  |  |  |  |  |  |  |  |  |  |  |  |  |  |  |  |  |  |  |  |  |  | |
|  |  |  | < .001 |  | — |  |  |  |  |  |  |  |  |  |  |  |  |  |  |  |  |  |  |  |  |  |  |  |  |  |  |  |  |  | |
| **E3** |  |  | 0.406 |  | 0.248 |  | — |  |  |  |  |  |  |  |  |  |  |  |  |  |  |  |  |  |  |  |  |  |  |  |  |  |  |  | |
|  |  |  | < .001 |  | < .001 |  | — |  |  |  |  |  |  |  |  |  |  |  |  |  |  |  |  |  |  |  |  |  |  |  |  |  |  |  | |
| **E4** |  |  | 0.377 |  | 0.281 |  | 0.603 |  | — |  |  |  |  |  |  |  |  |  |  |  |  |  |  |  |  |  |  |  |  |  |  |  |  |  | |
|  |  |  | < .001 |  | < .001 |  | < .001 |  | — |  |  |  |  |  |  |  |  |  |  |  |  |  |  |  |  |  |  |  |  |  |  |  |  |  | |
| **E6** |  |  | 0.492 |  | 0.323 |  | 0.342 |  | 0.443 |  | — |  |  |  |  |  |  |  |  |  |  |  |  |  |  |  |  |  |  |  |  |  |  |  | |
|  |  |  | < .001 |  | < .001 |  | < .001 |  | < .001 |  | — |  |  |  |  |  |  |  |  |  |  |  |  |  |  |  |  |  |  |  |  |  |  |  | |
| **E7** |  |  | 0.340 |  | 0.258 |  | 0.255 |  | 0.318 |  | 0.647 |  | — |  |  |  |  |  |  |  |  |  |  |  |  |  |  |  |  |  |  |  |  |  | |
|  |  |  | < .001 |  | < .001 |  | < .001 |  | < .001 |  | < .001 |  | — |  |  |  |  |  |  |  |  |  |  |  |  |  |  |  |  |  |  |  |  |  | |
| **E8** |  |  | 0.343 |  | 0.257 |  | 0.279 |  | 0.302 |  | 0.541 |  | 0.553 |  | — |  |  |  |  |  |  |  |  |  |  |  |  |  |  |  |  |  |  |  | |
|  |  |  | < .001 |  | < .001 |  | < .001 |  | < .001 |  | < .001 |  | < .001 |  | — |  |  |  |  |  |  |  |  |  |  |  |  |  |  |  |  |  |  |  | |
| **E9** |  |  | 0.364 |  | 0.371 |  | 0.333 |  | 0.331 |  | 0.529 |  | 0.524 |  | 0.603 |  | — |  |  |  |  |  |  |  |  |  |  |  |  |  |  |  |  |  | |
|  |  |  | < .001 |  | < .001 |  | < .001 |  | < .001 |  | < .001 |  | < .001 |  | < .001 |  | — |  |  |  |  |  |  |  |  |  |  |  |  |  |  |  |  |  | |
| **E10** |  |  | 0.368 |  | 0.293 |  | 0.342 |  | 0.362 |  | 0.498 |  | 0.390 |  | 0.445 |  | 0.643 |  | — |  |  |  |  |  |  |  |  |  |  |  |  |  |  |  | |
|  |  |  | < .001 |  | < .001 |  | < .001 |  | < .001 |  | < .001 |  | < .001 |  | < .001 |  | < .001 |  | — |  |  |  |  |  |  |  |  |  |  |  |  |  |  |  | |
| **E11** |  |  | 0.343 |  | 0.308 |  | 0.253 |  | 0.292 |  | 0.347 |  | 0.268 |  | 0.301 |  | 0.361 |  | 0.375 |  | — |  |  |  |  |  |  |  |  |  |  |  |  |  | |
|  |  |  | < .001 |  | < .001 |  | < .001 |  | < .001 |  | < .001 |  | < .001 |  | < .001 |  | < .001 |  | < .001 |  | — |  |  |  |  |  |  |  |  |  |  |  |  |  | |
| **E12** |  |  | 0.281 |  | 0.237 |  | 0.240 |  | 0.310 |  | 0.356 |  | 0.379 |  | 0.408 |  | 0.359 |  | 0.333 |  | 0.402 |  | — |  |  |  |  |  |  |  |  |  |  |  | |
|  |  |  | < .001 |  | < .001 |  | < .001 |  | < .001 |  | < .001 |  | < .001 |  | < .001 |  | < .001 |  | < .001 |  | < .001 |  | — |  |  |  |  |  |  |  |  |  |  |  | |
| **E13** |  |  | 0.323 |  | 0.236 |  | 0.263 |  | 0.272 |  | 0.510 |  | 0.500 |  | 0.595 |  | 0.502 |  | 0.427 |  | 0.381 |  | 0.436 |  | — |  |  |  |  |  |  |  |  |  | |
|  |  |  | < .001 |  | < .001 |  | < .001 |  | < .001 |  | < .001 |  | < .001 |  | < .001 |  | < .001 |  | < .001 |  | < .001 |  | < .001 |  | — |  |  |  |  |  |  |  |  |  | |
| **E14** |  |  | 0.369 |  | 0.219 |  | 0.281 |  | 0.246 |  | 0.394 |  | 0.407 |  | 0.463 |  | 0.548 |  | 0.477 |  | 0.407 |  | 0.423 |  | 0.611 |  | — |  |  |  |  |  |  |  | |
|  |  |  | < .001 |  | < .001 |  | < .001 |  | < .001 |  | < .001 |  | < .001 |  | < .001 |  | < .001 |  | < .001 |  | < .001 |  | < .001 |  | < .001 |  | — |  |  |  |  |  |  |  | |
| **E15** |  |  | 0.378 |  | 0.300 |  | 0.362 |  | 0.390 |  | 0.487 |  | 0.417 |  | 0.463 |  | 0.588 |  | 0.465 |  | 0.358 |  | 0.355 |  | 0.474 |  | 0.581 |  | — |  |  |  |  |  | |
|  |  |  | < .001 |  | < .001 |  | < .001 |  | < .001 |  | < .001 |  | < .001 |  | < .001 |  | < .001 |  | < .001 |  | < .001 |  | < .001 |  | < .001 |  | < .001 |  | — |  |  |  |  |  | |
| **S1** |  |  | 0.233 |  | 0.157 |  | 0.297 |  | 0.269 |  | 0.312 |  | 0.280 |  | 0.344 |  | 0.357 |  | 0.343 |  | 0.351 |  | 0.387 |  | 0.381 |  | 0.425 |  | 0.310 |  | — |  |  |  | |
|  |  |  | < .001 |  | 0.003 |  | < .001 |  | < .001 |  | < .001 |  | < .001 |  | < .001 |  | < .001 |  | < .001 |  | < .001 |  | < .001 |  | < .001 |  | < .001 |  | < .001 |  | — |  |  |  | |
| **S3** |  |  | 0.202 |  | 0.115 |  | 0.242 |  | 0.233 |  | 0.323 |  | 0.286 |  | 0.349 |  | 0.350 |  | 0.270 |  | 0.302 |  | 0.392 |  | 0.395 |  | 0.377 |  | 0.303 |  | 0.605 |  | — |  | |
|  |  |  | < .001 |  | 0.031 |  | < .001 |  | < .001 |  | < .001 |  | < .001 |  | < .001 |  | < .001 |  | < .001 |  | < .001 |  | < .001 |  | < .001 |  | < .001 |  | < .001 |  | < .001 |  | — |  | |

| **Table (A3): inter item correlations of items and their p-values in psychological health domain** | | | | | | | | | | | | | | | | | | | | | |
| --- | --- | --- | --- | --- | --- | --- | --- | --- | --- | --- | --- | --- | --- | --- | --- | --- | --- | --- | --- | --- | --- |
| Variable | | H1 | | H4 | | H8 | | H10 | | H11 | | H12 | | H13 | | H14 | | H16 | | H17 | |
| H1 |  | — |  |  |  |  |  |  |  |  |  |  |  |  |  |  |  |  |  |  |  |
|  |  | — |  |  |  |  |  |  |  |  |  |  |  |  |  |  |  |  |  |  |  |
| H4 |  | 0.470 |  | — |  |  |  |  |  |  |  |  |  |  |  |  |  |  |  |  |  |
|  |  | < .001 |  | — |  |  |  |  |  |  |  |  |  |  |  |  |  |  |  |  |  |
| H8 |  | 0.361 |  | 0.488 |  | — |  |  |  |  |  |  |  |  |  |  |  |  |  |  |  |
|  |  | < .001 |  | < .001 |  | — |  |  |  |  |  |  |  |  |  |  |  |  |  |  |  |
| H10 |  | 0.333 |  | 0.389 |  | 0.449 |  | — |  |  |  |  |  |  |  |  |  |  |  |  |  |
|  |  | < .001 |  | < .001 |  | < .001 |  | — |  |  |  |  |  |  |  |  |  |  |  |  |  |
| H11 |  | 0.310 |  | 0.413 |  | 0.429 |  | 0.554 |  | — |  |  |  |  |  |  |  |  |  |  |  |
|  |  | < .001 |  | < .001 |  | < .001 |  | < .001 |  | — |  |  |  |  |  |  |  |  |  |  |  |
| H12 |  | 0.233 |  | 0.349 |  | 0.331 |  | 0.425 |  | 0.663 |  | — |  |  |  |  |  |  |  |  |  |
|  |  | < .001 |  | < .001 |  | < .001 |  | < .001 |  | < .001 |  | — |  |  |  |  |  |  |  |  |  |
| H13 |  | 0.221 |  | 0.252 |  | 0.401 |  | 0.330 |  | 0.335 |  | 0.278 |  | — |  |  |  |  |  |  |  |
|  |  | < .001 |  | < .001 |  | < .001 |  | < .001 |  | < .001 |  | < .001 |  | — |  |  |  |  |  |  |  |
| H14 |  | 0.272 |  | 0.228 |  | 0.384 |  | 0.395 |  | 0.431 |  | 0.369 |  | 0.388 |  | — |  |  |  |  |  |
|  |  | < .001 |  | < .001 |  | < .001 |  | < .001 |  | < .001 |  | < .001 |  | < .001 |  | — |  |  |  |  |  |
| H16 |  | 0.241 |  | 0.269 |  | 0.414 |  | 0.376 |  | 0.331 |  | 0.245 |  | 0.438 |  | 0.356 |  | — |  |  |  |
|  |  | < .001 |  | < .001 |  | < .001 |  | < .001 |  | < .001 |  | < .001 |  | < .001 |  | < .001 |  | — |  |  |  |
| H17 |  | 0.222 |  | 0.216 |  | 0.317 |  | 0.320 |  | 0.233 |  | 0.168 |  | 0.318 |  | 0.376 |  | 0.528 |  | — |  |
|  |  | < .001 |  | < .001 |  | < .001 |  | < .001 |  | < .001 |  | 0.002 |  | < .001 |  | < .001 |  | < .001 |  | — |  |

| **Table (A4): inter item correlations of items and their p-values physical health domain** | | | | | | | | | | | |  |
| --- | --- | --- | --- | --- | --- | --- | --- | --- | --- | --- | --- | --- |
| Variable | | H2 | | H3 | | H5 | | H6 | | H7 | |  |
| H2 |  |  | — |  |  |  |  |  |  |  |  |  |
|  |  |  | — |  |  |  |  |  |  |  |  |  |
| H3 |  |  | 0.642 |  | — |  |  |  |  |  |  |  |
|  |  |  | < .001 |  | — |  |  |  |  |  |  |  |
| H5 |  |  | 0.390 |  | 0.319 |  | — |  |  |  |  |  |
|  |  |  | < .001 |  | < .001 |  | — |  |  |  |  |  |
| H6 |  |  | 0.396 |  | 0.325 |  | 0.296 |  | — |  |  |  |
|  |  |  | < .001 |  | < .001 |  | < .001 |  | — |  |  |  |
| H7 |  |  | 0.429 |  | 0.515 |  | 0.274 |  | 0.596 |  | — |  |
|  |  |  | < .001 |  | < .001 |  | < .001 |  | < .001 |  | — |  |
